# The challenges of mental health in Chilean university students

**DOI:** 10.3389/fpubh.2024.1297402

**Published:** 2024-03-12

**Authors:** Francisca Beroíza-Valenzuela

**Affiliations:** ^1^Universidad Metropolitana de Ciencias de la Educación, Santiago, Chile; ^2^Universidad de Chile, Santiago, Chile

**Keywords:** mental health, adolescence, social determinants, gender differences, COVID-19

## Abstract

Mental health is a crucial issue in Chile and worldwide, gaining even more relevance following social events in Chile in 2019, including the sociopolitical crisis and the COVID-19 pandemic. In Chile, over 20% of adolescents experience mental health problems such as anxiety, depression, and stress, with many going untreated due to limited access or the stigma associated with these issues. The COVID-19 pandemic has exacerbated this situation, with a 25% increase in the prevalence of anxiety and depression. The university population is particularly vulnerable to mental health challenges due to the unique pressures of the academic environment, including increased academic demands and the acquisition of university-related behaviors that can negatively impact physical and mental health, with notable gender differences. Effectively addressing these issues is essential for ensuring the emotional and psychological well-being of university students. Specific policies and programs are needed to address the mental health needs of university adolescents and ensure that they have access to mental health services required to navigate the challenges of daily life. A methodology reflective of the essayistic approach guides this exploration, which is characterized by critical reflection and structured argumentation.

## Introduction

The mental health is a crucial aspect of overall well-being, as acknowledged by researchers such as Chong et al. (1) and Ramsey et al. (2). In recent years, Chile has placed increasing emphasis on this issue, particularly in light of the sociopolitical crisis of 2019 (3) and the COVID-19 pandemic (4). The pandemic has underscored the significance of mental health and highlighted the need for specialized services worldwide (5). In Chile, mental health poses a significant challenge that impacts numerous individuals and communities nationwide, necessitating attention to ensure the emotional and psychological well-being (6). To address this issue comprehensively, it is crucial to understand the prevailing mental health challenges in Chile and to actively seek solutions to mitigate them. This entails recognizing the multifaceted nature of mental health issues, as well as acknowledging the unique sociocultural factors that contribute to their complexity in the Chilean context. By investigating the root causes and examining the specific challenges faced by diverse demographic groups, customized interventions and support systems can be developed.

The number of mental health-related claims has clearly increased in Chile (7, 8), this is compounded by the fact that the Agenda 2030, in its Sustainable Development Goal (SDG) number 3, stipulates that states must promote actions focused on the mental health and well-being of the population. (9). In this context, according to the “The Sustainable Development Goals Report 2022” regarding the progress of SDG 3, it highlights an increase in the prevalence of conditions such as anxiety and depression, with an up to 25% rise following the COVID-19 pandemic. In this regard, member countries are urged to take actions and allocate resources to mitigate this issue (10), as it affects the mobility of human capital for the progress and sustainable development of nations.

The university stage is a critical period in students’ developmental journey (11). During this time, university students are considered a psychologically vulnerable population as they assume new responsibilities and adapt to new environments and teaching styles (12–16). The increased academic demands, adherence to schedules, and engagement in risky behaviors such as the use of caffeine, tobacco, and alcohol (17, 18), contribute to the deterioration of both physical and mental health (19).

The academic environment is widely recognized as a significant stressor for university students (20). This acknowledgement is based on a complex array of obstacles that students encounter as they progress through their academic journey. The intense competition, demanding coursework, and relentless pressure to achieve academic excellence contribute to an environment where stress is omnipresent (21, 22). Academic stressors are multifaceted and encompass factors such as challenging assignments, tight deadlines, and expectations of maintaining high grades. These stressors can result in the development of mental health problems including depression, anxiety, and stress symptoms (23). The competitive nature of academic settings often leaves students struggling to meet high expectations, both self-imposed and external. Consequently, the pursuit of academic excellence can inadvertently foster a culture of perfectionism, where fear of failure and the constant need for validation create an environment that exacerbates mental health challenges.

There is a trend among college students to avoid seeking psychological assistance, a phenomenon that might be influenced by cultural beliefs surrounding psychotherapy and psychological factors such as expectations (24, 25). Students’ reluctance to seek mental health support can be linked to cultural norms that stigmatize discussions on mental health or therapy. In certain cultural contexts, some individuals may believe that seeking help for mental health concerns signifies a lack of strength or failure to cope (26). The societal stigma surrounding mental health problems may discourage students from openly acknowledging their struggles or seeking professional support (27). Additionally, students’ personal expectations and perceptions of their mental well-being can significantly influence their willingness to seek assistance. Some students may have overly optimistic views of themselves, believing that they should be able to handle all aspects of their lives without outside help (28). This self-imposed pressure not to seek help can be exacerbated by fear of judgment or concerns about how seeking psychological support might be perceived by others (29). These factors could lead to worsening psychological situations by not receiving timely support in the early stages, where it is possible to prevent, intervene, and address mental health concerns (30). Furthermore, most students and the educational community are unfamiliar with healthy living standards, which can negatively affect the psychological and physical health of members.

In the academic environment, significant dissimilarities exist in mental health symptomatology between men and women (31). Moreover, there are disparities in the inclination of men and women to seek professional assistance for mental health issues (32). This may be due to adherence to specific traits such as emotional control, self-sufficiency, dominance, masculine rigidity, and resistance to attributes considered feminine (33, 34). Recognizing and understanding these gender-specific dynamics is crucial for devising targeted interventions that acknowledge the unique needs and challenges faced by male and female students. Failure to consider these nuances may result in the development of interventions that do not effectively address the diverse mental health experiences within the university population. For instance, international studies indicate a gender gap in mental health, with a higher prevalence of mental health issues among women (35, 36). Similarly, this trend was observed in Chile, where studies have shown an increase in psychological distress in women during the pandemic, including anxiety, depression, and stress (37), Research has also found a higher prevalence of depressive symptoms in women (23.2%) than in men (13.4%). This, combined with low social support and stressful life events, constitutes the combined effect of socioeconomic and gender inequalities (38). Regrettably, little is known about the extent to which the gender gap affects these figures, even though it shapes individual and social behaviors. Therefore, it is essential to study these issues to understand the phenomenon and encourage the government to formulate public policies that contribute to the well-being of individuals, enabling them to develop their competencies and skills optimally.

Among the numerous challenges university students encounter, mental health issues are among the most prevalent, with studies indicating a rate of 20–30% of students experience such difficulties, particularly depression and anxiety disorders (39, 40). Furthermore, these rates are even higher among individuals from lower socioeconomic backgrounds (41). It is important to note that these statistics were obtained prior to the COVID-19 pandemic, which has likely exacerbated the situation and further strained the resources of student affairs offices. To develop effective prevention and treatment policies, it is crucial to consider the unique needs of the community.

Among the many challenges faced by university students, it is noteworthy that approximately one in five students experience suicidal ideation and one in 15 have attempted suicide (42). Moreover, these figures are expected to have increased since then. Furthermore, substance abuse exacerbates these conditions, adding another layer of complexity to the situation. Substance abuse is often viewed as part of university culture (43), with over half of the students reporting the use of alcohol and drugs (44). Given that these factors contribute to increased mental health symptoms (45, 46), it is crucial to develop tailored management and prevention programs. Additionally, academic stress is a significant factor in the university context that affects students’ mental health. The primary sources of stress include academic workload and the amount of time dedicated to studying (47). Importantly, this factor exacerbates eating disorders and substance abuse (48). These four elements are critical for understanding the areas in which intervention is necessary for higher education institutions to address the mental health challenges faced by students.

The purpose of this essay was to examine the mental well-being of university students in Chile and explore the influence of the COVID-19 pandemic, gender disparities, and the resumption of in-person education on their mental health. This analysis highlights the significance of addressing this issue for the country’s socioeconomic progress, as higher education is vital for social mobility and the development of sophisticated human resources. This work presents a perspective in this field, considering the sociopolitical implications and the post-pandemic context. Moreover, specific recommendations are suggested for establishing a healthy environment for university students. The methodology used in this study adheres to the characteristics of an essay that entails critical reflection and structured argumentation. To substantiate these arguments, a comprehensive review of pertinent literature was conducted. The essay also encourages dialog and exploration of diverse viewpoints, reflecting the discursive nature of this literary form.

## Sociopolitical considerations of mental health in university adolescence

The legislation in Chile regarding the mental health of university adolescents is guided by the Mental Health Plan, which aims to ensure the right to mental health protection and promote psychological and social well-being across the lifespan through the prevention, promotion, treatment, and rehabilitation of individuals with mental disorders (Ministerio (49)). Additionally, this legislation is supported by the Higher Education Law, which includes regulations for promoting a safe and healthy environment for students as well as supporting mental health promotion programs in university institutions (50).

The lack of a dedicated institution to address adolescent mental health concerns can have detrimental consequences for the overall welfare of young individuals. Given the psychological ramifications of the pandemic on the population, it is vital to underscore the absence of a clear political plan to address this urgent social need, given the psychological ramifications of the COVID-19 pandemic on the population (51). It is imperative for the state and society as a whole to assume responsibility for comprehensively addressing this issue and catering to the specific requirements of adolescents.

Coordinated political action is essential to ensure access to mental healthcare services for adolescents across the nation. Various social determinants can impact mental health, including demographic factors, such as gender, ethnicity, and age gaps, as well as economic aspects, such as poverty, insecurity, and job precariousness. Additionally, support networks in the neighborhood environment, such as social cohesion, violence, and the quality of infrastructure, can also play a significant role, as can elements linked to climatic events and social and cultural aspects such as education, social cohesion, social capital, culture, and social class (52, 53). Mental health issues can be explained by variability in living conditions, many of which result from social inequities (54). Therefore, intersectoral coordination is necessary to ensure universal protection as a regulatory right framework.

## Post-COVID-19 pandemic: diagnosis of mental health in Chilean university students

The global mental Health Post-Coronavirus Pandemic is an issue that affects various population groups. According to an international study conducted by researchers from the Queensland Mental Health Research Center in the United States and published in the esteemed journal The Lancet, depressive disorders experienced an increase of 27.6%. The prevalence of depressive disorders among women was higher (29.8%) than that among men (24.0%). Similarly, women have experienced a higher increase in the prevalence of anxiety disorders (27.9%) than men (21.7%) (55). The study conducted by researchers at Boston University and published in the Journal of Affective Disorders between 2013 and 2020 revealed sex differences in relation to clinical cases of mental health. The researchers found that depression increased by almost 135% over 8 years, while anxiety increased by 110%. Furthermore, during the peak years of the pandemic–2020-2021, the prevalence of mental health problems increased by 60% (56).

Various international studies have reported an increase in mental health disorders among university students who, due to the pandemic, experience high levels of anxiety and depression (57, 58), suicidal ideation (59, 60), stress, fear (61), emotional, learning, financial, social, and technological impacts (62), changes in eating habits, sleep, lifestyle, and behavior (63), and nightmares (64). These factors impair academic performance (65). Studies indicate that women have reported worse mental health than men (66), highlighting gender differences in mental health, which is a matter of great concern, as it does not contribute to the overall well-being of women and hinders their emotional and social progress and development (67).

Data for the year 2022 in Chile revealed a significant deterioration in mental well-being. The prevalence of mental health issues increased by 21.1% compared to the previous year, with generalized anxiety disorders increasing by 27.5% in both men and women but at a higher rate among women (36.6%). Depression, however, saw an increase of 16.4%, and women once again experienced a higher prevalence of 22.4% (68). Studies conducted before the COVID-19 pandemic suggested that mental health disorders in university students could be more complex than those in the general population (12, 69). These issues in the university population contribute significantly to the global burden of disease and can impede young individuals’ ability to complete age-relevant tasks during critical periods of their development (70), This period is considered complex in terms of identity exploration (71, 72).

A 2018 study conducted at three universities belonging to the Council of Rectors revealed a distressing situation, with over 45% of clinical cases involving depression, anxiety, and stress frequently presenting in a comorbid state. Women in this study had higher levels of anxiety and stress. Approximately 5% of university students reported suicidal ideation and 14% exhibited risky eating behaviors, with a higher prevalence among women. Additionally, 50% of the participants reported symptoms of insomnia, while 42% experienced daytime hypersomnia (17).

In light of the pandemic, a study carried out in September 2020 among students at the University of Los Andes revealed a substantial prevalence of mental health clinical conditions, with alarming rates of stress (54.6%), anxiety (37.9%), depression (37.1%), and suicide risk (20.4%) (73). These findings underscore the need for a comprehensive examination of clinical symptoms associated with mental health disorders in university students. However, merely identifying the frequency and severity of these symptoms is insufficient for developing tailored intervention strategies. In addition to eating disorders, self-destructive behaviors, problematic substance use, and academic stress (74), there are affective and social spaces that serve as protective factors or early indicators that must be considered. For instance, assessing the positive and negative affect of students can provide valuable information about whether they engage in rewarding and energetic experiences or struggle with an unpleasant and distressing sense of participation (75). While this in and of itself is not a problem, a high presence of negative affect is evidence of a contextual issue within the university community that needs to be addressed before these affects evolve or contribute to the emergence of severe symptomatology.

## Discussion

The mental health of college students is a significant issue in the contemporary society. During the transitional period between childhood and adulthood, young people undergo both physical and emotional changes, which can lead to the development of mental disorders that can affect their well-being and future prospects (76). To undertake this issue, it is crucial to establish strategies for the prevention, detection, and early treatment of these disorders. Failure to do so may result in negative consequences for the lives of adolescents nationwide. Maintaining good mental health involves developing mental health literacy; adopting a positive attitude toward mental disorders; cultivating self-perception and values that promote a meaningful life; developing cognitive skills for decision-making and problem-solving; demonstrating adaptability; focusing on emotional states, behaviors, and self-management strategies; developing social skills; maintaining physical and sexual health; nurturing family and meaningful relationships; possessing a sense of purpose in life; and achieving a good quality of life (77). These domains can serve as a foundation for standardizing future public policy actions to promote good mental health.

One of the main obstacles hindering the resolution of adolescent mental health issues is the social stigma associated with mental disorders, as evidenced by Gerlinger et al. (78) and Haugen et al. (79). Often, these young individuals shy away from seeking help because of fear of rejection or stigmatization. Therefore, fostering a culture of respect and acceptance for those affected by mental illness is of utmost importance. It is essential that adolescents realize that they are not alone, and that resources and professionals are available to assist them.

Education is a vital factor for adolescents’ emotional well-being. It is crucial to teach young individuals essential emotional skills from a tender age to enable them to manage their emotions in a healthy manner. Moreover, incorporating mental disorder prevention programs in universities and equipping teachers with the ability to detect possible mental health issues are essential. Accessible and affordable mental healthcare services for all adolescents are also necessary. For this, community engagement is necessary to ensure access to mental healthcare services and to implement measures focused on this area (80, 81). Such initiatives promote a culture of respect and acceptance, while providing accessible education and resources for all young people, allowing them to lead a fulfilling and satisfying life. To address these challenges, a comprehensive and collaborative response that involves multiple stakeholders is necessary. Convincing evidence indicates that policymaking at all levels of government and across all sectors can significantly impact mental health outcomes, emphasizing the importance of inter-sectoral work.

The present investigation comprehensively explored the mental health of university students in Chile. This is a critical component of overall well-being. Despite the limitations imposed by the essay format on the research methodology, it is essential to explore the importance of addressing students’ mental health and engaging in extensive discourse on the subject. This would facilitate a deeper understanding of the issue, although the study relied on subjective interpretations and critical evaluations.

## Data availability statement

The original contributions presented in the study are included in the article/supplementary material, further inquiries can be directed to the corresponding author.

## Author contributions

FB-V: Conceptualization, Data curation, Formal analysis, Funding acquisition, Investigation, Methodology, Project administration, Resources, Software, Supervision, Validation, Visualization, Writing – original draft, Writing – review & editing.
